# Targeting mTOR for the treatment of AML. New agents and new directions

**DOI:** 10.18632/oncotarget.290

**Published:** 2011-06-15

**Authors:** Jessica K. Altman, Antonella Sassano, Leonidas C. Platanias

**Affiliations:** ^1^ Robert H. Lurie Comprehensive Cancer Center and Division of Hematology-Oncology, Northwestern University Medical School, and Jesse Brown VA Medical Center, Chicago, IL

**Keywords:** Acute myeloid leukemia, mTOR, TORC2, TORC1, rapamycin, kinase, signaling, chemotherapy, cell survival

## Abstract

Despite recent advances in the field, the treatment of patients with acute myeloid leukemia (AML) remains challenging and difficult. Although chemotherapeutic agents induce remissions in a large number of patients, many of them eventually relapse and die. A major goal for the development of new approaches for the treatment of AML is to enhance the antileukemic effects of standard chemotherapeutics and to design effective combinations targeting non-overlapping cellular pathways. The PI3K/Akt/mTOR signaling pathway plays a critical role in survival and growth of malignant cells and its targeting has been the focus of extensive work and research efforts over the last two decades. It now appears possible that a major limitation of the first generation of mTOR inhibitors can be overcome by a new class of catalytic inhibitors of mTOR. There is emerging evidence that such compounds target both TORC1 and TORC2 and elicit much more potent responses against early leukemic precursors *in vitro*. In addition, recent studies have shown that combinations of such agents with cytarabine result in enhanced antileukemic responses in vitro, raising the prospect and potential of use of these agents in combination regimens for the treatment of AML.

## INTRODUCTION

Untreated, acute myeloid leukemia (AML) is a fatal hematological malignancy. Although remissions can be achieved with intensive chemotherapy, the disease relapses in a large number of cases and progression and death frequently occurs [1-5]. The current treatment strategies, involving combinations of cytarabine with an anthracycline, result in substantial toxicity and morbidity. This is a particularly serious problem in the case of older adults with the disease, who frequently have less favorable outcomes than younger patients. Undoubtedly, there is an urgent need for new treatments and therapeutic approaches. AML appears to result from mutations of key genes that ultimately lead to deregulation and constitutive activation of cellular cascades that promote cell growth and mediate anti-apoptotic and pro-survival responses. Such changes result in deregulation of normal hematopoiesis and promote malignant transformation leukemogenesis [6-10].

A major problem in efforts to treat and cure AML is the inability to efficiently target and eliminate leukemia initiating cells (LICs), which are the cells that initiate and maintain the leukemic phenotype [10, 11]. The majority of LICs are quiescent and therefore not sensitive to various chemotherapeutic drugs that target and kill rapidly dividing cells [12, 13]. This fact explains in part the difficulty in eliminating leukemia with chemotherapy and the relapses seen in the majority of patients, despite initially achieving complete responses with classical chemotherapy regimens. Aberrant activation of pro-survival signaling cascades in leukemia stem cells and early committed leukemic precursors may also act protectively and promote their survival, providing a potential therapeutic outlet and elements that can be targeted for the treatment of leukemias [12, 14].

## THE PI3’ KINASE/mTOR PATHWAY

The PI3’ kinase/AKT/mTOR pathway is a key regulatory network of signaling cascades in mammalian cells, whose coordinated operations regulate and promote important cellular activities and functions [15, 16]. Via controlling activation of the mTOR kinase and its downstream effectors, this cellular network ultimately regulates mRNA translation of genes that encode for pro-oncogenic proteins and, thus, promote malignant cell survival [15-18]. Interestingly, this network of signals is also engaged and activated by growth suppressive cytokines such as interferons (IFNs) [19-21], suggesting a competition between factors that suppress growth and mitogenic signals for the use and regulation of this pathway. PI 3’ kinase (PI3’K) is a lipid kinase that controls formation of distinct signaling complexes on the membrane of cells [22]. Activation of PI3’K leads to engagement of the kinase PDK1 which subsequently phosphorylates AKT on threonine 308 (Thr 308), ultimately leading to engagement and activation of AKT [23], which in turn phosphorylates many and activates multiple downstream substrates and effectors, leading to the generation of signals that promote survival and proliferation [24, 25].

As aberrant activation of the PI3’K/AKT/mTOR pathway promotes malignant cell proliferation and survival [15, 26, 27], several studies have sought to examine the implications of constitutive activation of this pathway in tumorigenesis. There is now extensive evidence that deregulation of this pathway contributes to the tumorigenic potential, a more aggressive phenotype and poorer prognosis in several malignancies [28-30]. In addition, activation of this pathway has been associated with chemotherapy resistance [31, 32], underscoring the importance of this signaling cascade as a therapeutic target for the treatment of various tumors. For all these reasons, there has been a major interest in the development of pharmacologic inhibitors of the PI3K/AKT/mTOR pathway for various solid tumors and hematological malignancies, which has further intensified after the detailed mapping and characterization of the pathway the last several years.

**Figure 1:**
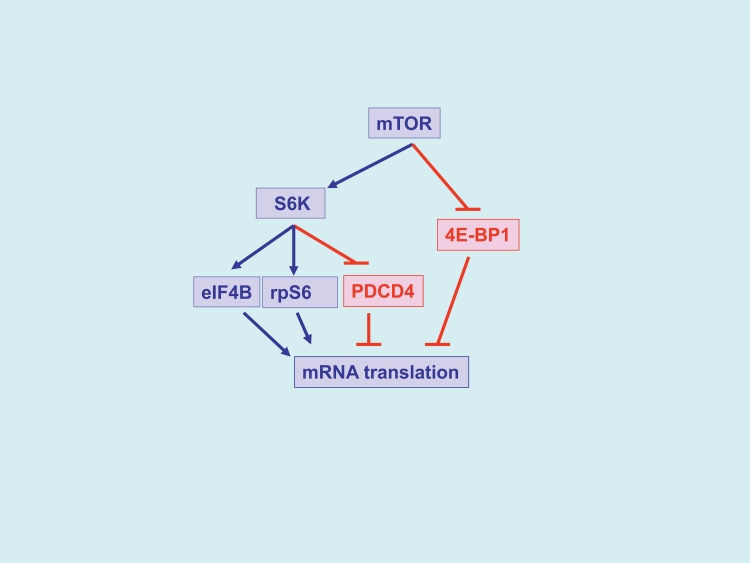
TORC1 and TORC2 complexes and inhibitory effects of different mTOR inhibitors The rapalogs (shown in black), inhibit selectively TORC1, while the catalytic TOR inhibitors, (shown in red), inhibit both TORC1 and TORC2.

**Figure 2:**
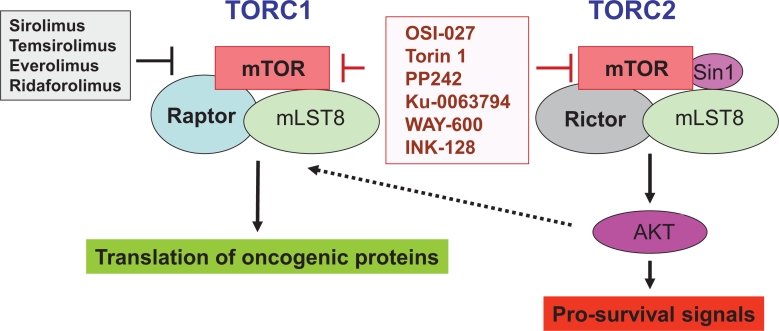
Effector elements downstream of mTOR that exhibit positive (in blue) or negative (in red) effects on mRNA translation

The mammalian target of rapamycin (mTOR) is a central element of the pathway and a key kinase activated downstream of PI3K/AKT. This kinase was originally identified in yeast [33], and subsequent work established that it is conserved in eukaryotic organisms. mTOR is present in two distinct and functionally diverse cellular complexes: TORC1 and TORC2 (Fig. 1). Each of these 2 complexes have common and distinct subunits and effectors and ultimately engage different downstream elements and activate distinct effector pathways. As shown in Fig. 1, the interaction of mTOR with Raptor (regulatory-associated protein of mTOR) defines the TORC1 complex [34-37]. TORC1 is generally perceived as rapamycin-sensitive and beyond mTOR and Raptor, it also contains mLST8 [35-37]. The two major substrates for TORC1 are the S6 kinase (S6K) and the translational repressor 4E-BP1, which binds to and negatively controls the function of the eukaryotic initiation factor 4E (eIF4E) [16, 19, 35-39]. After its phosphorylation/activation by mTOR, S6K regulates downstream engagement of two major substrates, the S6 ribosomal protein (rpS6) and the eukaryotic initiation factor 4B (eIF4B) [16, 35-39] (Fig.2). In addition, there is recent evidence that it phosphorylates and negatively regulates the expression of PDCD4 (Fig. 2), a tumor suppressor protein with inhibitory activities on cap-dependent translation via its ability to block the function of the translation initiation factor eIF4A and the integration of eIF4A into the eIF4F complex [40-44]. This protein undergoes phosphorylation by S6K, followed by degradation by the ubiquitin ligase βTRCP (45), suggesting a mechanism by which the mTOR pathway may be targeting and inhibiting tumor suppressor elements with regulatory effects on mRNA translation. In addition, to regulating activation of S6K, the mTORC1 complex is responsible for phosphorylation of the (eIF4E)–binding proteins (4E-BP) 1 and 2 on several sites, leading to their de-activation and detachment from eIF4E [19, 35-39]. Such dissociation allows eIF4E activation, which is a critical event for the initiation of mRNA translation by oncogenic proteins in eukaryotes.

In contrast to TORC1, whose major function is control of signals for the initiation of mRNA translation, the TORC2 complex plays a different role in normal and malignant cells. The TORC2 complex includes mTOR, Rictor (rapamycin-insensitive companion of mTOR), SIN1, and mLST8 [35-37, 46-48]. The primary function of TORC2 is the control of phosphorylation of AKT on Ser 473 [35-37], a site whose phosphorylation is essential for activation of AKT resulting in induction of its kinase domain. Beyond AKT, additional substrates for TORC2 activity have been identified, including PKCα, [49-51] and SGK1 [52, 53]. Notably it was recently shown that PKCα gene expression is inducible in AML cells resistant to chemotherapy [54].

Because of the high relevance of the mTOR pathway in malignancies, first generation mTOR inhibitors, including rapamycin and related drugs (rapalogs) have been tried extensively in various clinical contexts for the treatment of tumors of diverse cellular origin. Two rapalogs, temsirolimus (CCI-779) and everolimus (RAD001) have shown major activity and have been approved by the FDA for the treatment of renal cell carcinoma [55, 56]. Since then extensive clinical efforts have been ongoing in attempts to evaluate the clinical activity of the three major rapalogs (everolimus, temsirolimus and ridaforolimus) in the treatment of various solid tumors and hematological malignancies [57-60].

## TARGETING mTOR IN AML

The simultaneous deregulation of pathways that control both transcription and mRNA translation of genes encoding for oncogenic proteins appears to play key roles in the pathogenesis and pathophysiology of AML. There has been extensive evidence that the PI3’K/AKT/mTOR pathway is aberrantly activated and deregulated in AML [61]. There is also some evidence that constitutive PI 3’K activation in AML is mainly due to the activity of the PI3K p110δ isoform [62, 63]. In one study, a large percentage of samples from patients with AML were found to have constitutive AKT activation [64]. In addition, the AKT pathway was among the signaling cascades whose simultaneous activation with other pathways, such as PKCα and ERK, was found to confer a poor prognosis in AML [65]. Other recent studies used proteomic analysis or single-cell network profiling (SCNP) with flow cytometry, to predict the likelihood of response to induction chemotherapy for patients with AML [66, 67]. Remarkably, lack of response to induction chemotherapy in patients older than 60 years or patients with secondary AML was associated with increased phosphorylation of AKT induced by FLT-3 ligand [67].

Recognition of aberrations in the AKT/mTOR pathway has led to clinical trials with rapalogs in AML. Recher et al showed that rapamycin resulted in blast clearance in some patients with AML. However, the length of response was limited and not all patients responded [68]. There has been also some evidence that rapamycin and etoposide exhibit synergistic/enhancing effects on AML cells *in vitro* and in AML mouse models *in vivo* [69]. However, when a clinical trial involving the addition rapamycin to salvage chemotherapy (mitoxantrone, etoposide, and cytarabine) for the treatment of relapsed and refractory AML was performed, the authors failed to observe synergistic activity by the combination [70].

## NEW APPROACHES TO TARGET TORC1 AND TORC2 COMPLEXES IN AML

Although approaches to optimize the administration of rapalogs with chemotherapy [71], in various settings are still being examined, the use of these agents has several limitations as discussed above. To overcome the limitations of the rapalogs, extensive efforts over recent years have been focused on the design and clinical development of agents that are catalytic inhibitors of mTOR and in addition to TORC1 suppress TORC2, or other agents that simultaneously target the PI3’K/AKT pathway. Several pan PI3K/AKT/mTOR inhibitors and dual TORC inhibitors have been developed and are being exploited [72-79]. Such efforts have also been extended to determine the effects of such compounds on leukemias. Recent studies demonstrated that the dual TORC1/TORC2 inhibitors PP242 [80] or OSI-027 [81] are potent suppressors of both TORC1 and TORC2 activities in BCR-ABL transformed cells. These catalytic inhibitors were shown to elicit potent antileukemic effects *in vitro* [80, 81] and ***in vivo*** [81] on CML or Ph+ ALL cells, including cells expressing the T315I BCR-ABL mutation, which is resistant to the kinase inhibitors currently approved for use in the treatment of CML and Ph+ ALL (imatinib mesylate, nilotinib, dasatinib).

The potent suppressive effects of dual TORC1/TORC2 inhibitors on BCR-ABL-transformed cells, have raised the possibility that such agents may have activity in other leukemias and prompted us to perform additional studies to examine the spectrum of the antileukemic properties of OSI-027 in AML. In recently published work [82], we examined the effects of dual TORC1/2 inhibition on various elements of the mTOR pathway in different AML cell lines and primary leukemia blasts from AML patients and compared them to the effects of the classic mTOR inhibitor rapamycin. As expected, only OSI-027 blocked TORC2-specific cellular events in AML cells, such as phosphorylation of AKT on Ser473 [82]. On the other hand, both OSI-027 and rapamycin were potent suppressors of the activation of the S6 kinase and the downstream phosphorylation of its target, S6 ribosomal protein [82] Importantly, phosphorylation of 4E-BP1 on Thr 37/46 was blocked by OSI-027, but not rapamycin, indicating that such phosphorylation is a rapamycin-insensitive cellular event in AML cells (79). This is consistent with the emerging evidence in other systems for rapamycin-insensitive TORC1-mediated signals [83, 84]. Our studies also established that OSI-027 is a potent suppressor of primitive leukemic precursors (CFU-L) from AML patients. Such effects were much more potent than the effects of rapamycin analyzed in parallel [82]. In addition, OSI-027 enhanced the inhibitory effects of low-dose cytarabine (Ara-C), suggesting that combinations of dual TORC1/2 inhibitors with chemotherapy may provide an approach to enhance antileukemic responses of chemotherapy [82].

Altogether, the results of such work raise the prospect of future clinical trials using dual TORC1/TORC2 inhibitors for the treatment of AML. Beyond OSI-027 there are additional TORC1/2 inhibitors in clinical or pre-clinical development [73-77, 85] that may be good candidates for such studies. Another potential approach to generate antileukemic responses by complete inhibition of the mTOR pathway would be to block the PI3’K/AKT axis [86]. In fact, approaches to simultaneously block PI3’K and mTOR have been developed [87]. NVPBEZ235 is a molecule that inhibits the PI3’K and also both TORC1 and TORC2 complexes [88]. Recent studies using this agent in AML have demonstrated potent inhibitory effects on PI3’K and TORC1/TORC2 complexes, including rapamycin-insensitive TORC1. It was also found to inhibit rapamycin-insensitive phosphorylation sites in 4E-BP1 [89]. Such potent effects were associated with decreased cell proliferation and survival of leukemia cells and suppressed leukemic progenitor clonogenicity [89], raising the prospect of using such pan P13’K/AKT/mTOR inhibitors as a potential future approach for the treatment of AML.

## SUMMARY

While inhibiting mTOR is a promising strategy for the treatment of malignancies, agents that selectively target TORC1 (rapalogs) have limited clinical activity and are unlikely to have major impact in the treatment of AML. The development of selective ATP-catalytic inhibitors, which have the capacity to block the functions of both TORC1 and TORC2 has resulted in new momentum in the research field of mTOR targeting in AML and is igniting important work with major therapeutic implications. Approaches to overcome the limitations of rapalogs for the treatment of leukemias are now possible, using either dual TORC1/2 inhibitors or pan–PI3K-TORC1/2 inhibitors. Our recent studies have established that beyond exhibiting potent antileukemic effects, dual TORC1/2 catalytic inhibition enhances the effects of cytarabine on primitive leukemic precursors from AML patients. These studies are very encouraging and suggest a potential role for these agents in the treatment of AML patients. They also raise the possibility that combinations of dual TORC1/2 inhibitors with chemotherapeutic agents may provide a novel approach to target leukemic initiating stem cells and increase the probability of cure for AML patients.
